# The Role of Autophagy in Chloroplast Degradation and Chlorophagy in Immune Defenses during *Pst* DC3000 (*AvrRps4*) Infection

**DOI:** 10.1371/journal.pone.0073091

**Published:** 2013-08-30

**Authors:** Junjian Dong, Wenli Chen

**Affiliations:** 1 MOE Key Laboratory of Laser Life Science & Institute of Laser Life Science, College of Biophotonics, South China Normal University, Guangzhou, Guangdong, China; 2 College of Life Science, Guangdong Key Laboratory of Biotechnology for Plant Development, South China Normal University, Guangzhou, Guangdong, China; Justus-Liebig-University Giessen, Germany

## Abstract

**Background:**

Chlorosis of leaf tissue normally observed during pathogen infection may result from the degradation of chloroplasts. There is a growing evidence to suggest that the chloroplast plays a significant role during pathogen infection. Although most degradation of the organelles and cellular structures in plants is mediated by autophagy, its role in chloroplast catabolism during pathogen infection is largely unknown.

**Results:**

In this study, we investigated the function of autophagy in chloroplast degradation during avirulent *Pst* DC3000 (*AvrRps4*) infection. We examined the expression of defensive marker genes and suppression of bacterial growth using the electrolyte leakage assay in normal light (N) and low light (L) growing environments of wild-type and *atg5-1* plants during pathogen treatment. Stroma-targeted GFP proteins (CT-GFP) were observed with LysoTracker Red (LTR) staining of autophagosome-like structures in the vacuole. The results showed that *Arabidopsis* expressed a significant number of small GFP-labeled bodies when infected with avirulent *Pst* DC3000 (*AvrRps4*). While barely detectable, there were small GFP-labeled bodies in plants with the CT-GFP expressing *atg5-1* mutation. The results showed that chloroplast degradation depends on autophagy and this may play an important role in inhibiting pathogen growth.

**Conclusion:**

Autophagy plays a role in chloroplast degradation in *Arabidopsis* during avirulent *Pst* DC3000 (*AvrRps4*) infection. Autophagy dependent chloroplast degradation may be the primary source of reactive oxygen species (ROS) as well as the pathogen-response signaling molecules that induce the defense response.

## Introduction

Plants have developed a multilayered systematic immune system that is activated in response to a pathogen attack. In general, plants adopt numerous defense responses that deploy two primary levels of defense, i.e., basal defense and resistance (R) gene-mediated defense [1]. However, some pathogens have developed the ability to evade basal defenses by secreting virulence effectors. In response to this evasion, plants with R genes have evolved. The R genes can directly or indirectly recognize specific microbial virulence effectors and activate a second level of defense for effector-triggered immunity (ETI) [1,2]. ETI against pathogens is accompanied by the burst of reactive oxygen species (ROS), the up-regulation of pathogenesis-related genes, the production of several antimicrobial compounds, and the elicitation of hypersensitive response (HR) at sites of infection [3,4]. There is increasing evidence that suggests that the chloroplast plays a significant role during ETI [2].

The chloroplast is one of two primary sources of ROS production in the plant cell [5], and is responsible for producing the pathogen-response signaling molecules salicylic acid (SA) and jasmonic acid (JA). The another primary source of ROS is the membrane-associated NADPH oxidase complex [2,6]. Recently, it has been shown that chloroplastic NADPH oxidase-like activity-mediates hydrogen peroxide generation in the chloroplast [7]. ROS represent primary defense against pathogens, triggering secondary messengers for Systemic Acquired Resistance (SAR). It is commonly observed that chlorosis of plant tissue is induced by pathogen infection and this may be due to targeted degradation or disruption of the chloroplasts [4,8]. During pathogenic stress, plants likely mobilize needed nitrogen, trigger the burst of ROS and generate pathogen-response signaling molecules from within plant cell chloroplast to other organelles or surrounding cells [9]. Although most degradation of the organelles and cellular structures in plants is mediated by autophagy, evidence shows that autophagy plays a role in chloroplast degradation during senescence [10,11]. The role of autophagy in the degradation of chloroplasts during pathogen attack is largely unknown. Autophagy is regarded as a protection mechanism induced by pathogen infection during plant immunity [12,13]. It has been postulated that autophagy may have a function in the degradation of chloroplasts during some plant–pathogen *R-Avr* gene interactions.

Because over half of total leaf nitrogen is distributed into the mesophyll chloroplast proteins [14], chloroplasts and chloroplasts proteins are frequently attacked by pathogens. Some evidence shows that some pathogenic virulence effectors may restrain defense signaling initiated from chloroplasts [15–17]. For instance, the pathogenic effector Hopl1 localizes to chloroplasts, the site of SA synthesis, which causes thylakoid reconstitution and inhibition of SA synthesis [17]. However, plants have developed numerous defense responses against pathogen attack. If virulence effectors are perceived by a specific R gene, they have to be acted as avirulent factors, e.g., *AvrRps4* avirulent gene is recognized by *Rps4* R gene [4,18]. R genes then activate the second level of defense, ETI, against invading pathogens. Hofius et al. (2009) recently showed that autophagy has an immune enhancing function by triggering a rapid defense response and death promoting function through plant–pathogen *Rps4*-*AvrRps4* gene interaction [4,19]. While pathogens probably disrupt entire chloroplasts or chloroplast proteins, it is possible that autophagy, triggered by the R gene *Rps4* or the defense regulator *EDS1/PAD4*, is involved in the removal of destructed organelles, mobilizing needed nitrogen or triggering the generation of ROS, SA, and JA.

## Results

### Avirulent Pst DC3000 (AvrRps4) Infection Induces Spreading Disease Symptoms in Arabidopsis Leaves

**Figure 1:**
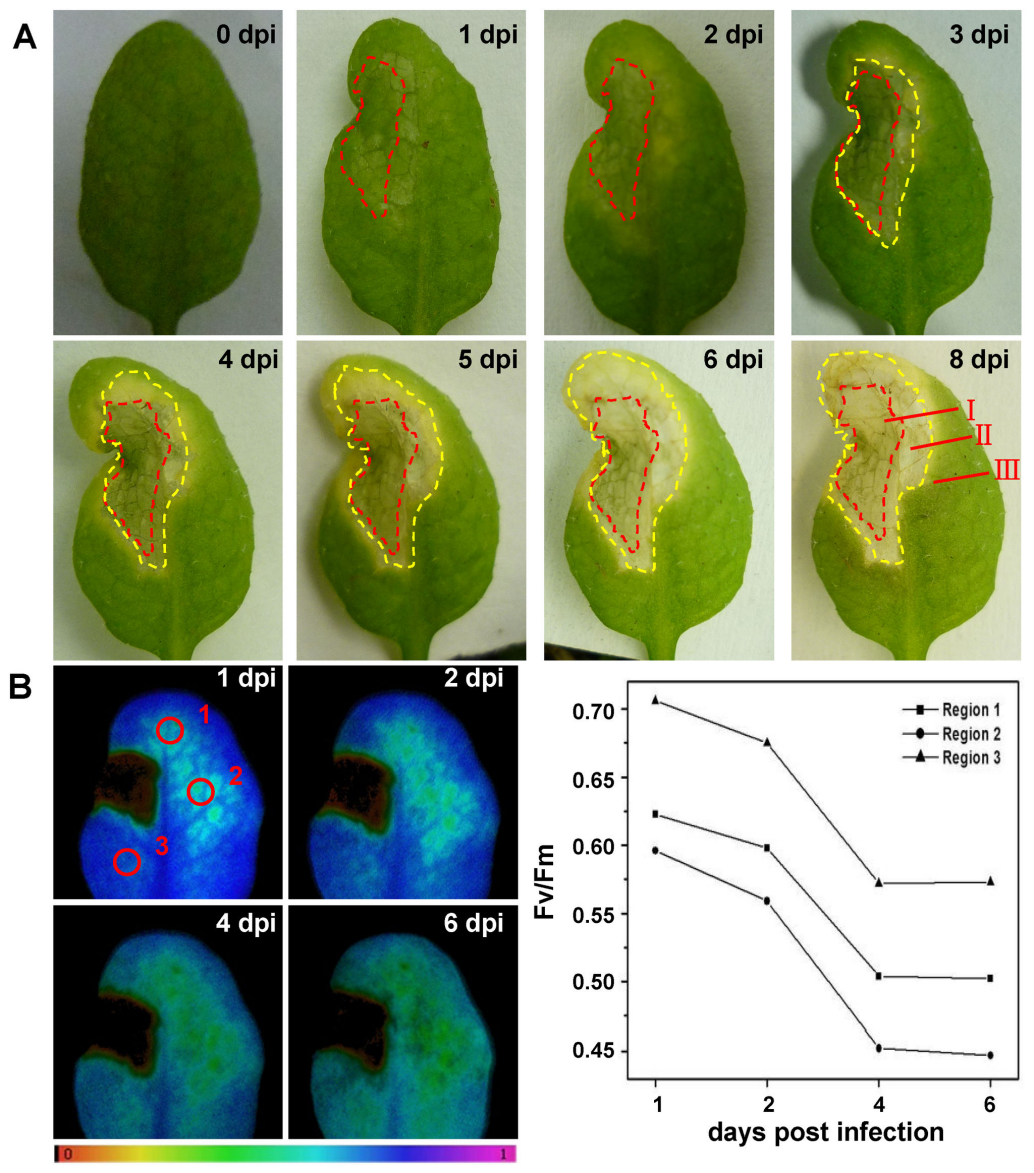
The effect of local infection with avirulent *Pst* DC3000 (*AvrRps4*) on phenotype and chlorophyll fluorescence parameter. A. *Pst* DC3000 (AvrRps4) induced HR lesions are contained in 4-week-old wild-type (Col-0). Representative images of disease symptoms were photographed at 0, 1, 2, 3, 4, 5, 6 and 8 dpi after local infection with avirulent *Pst* DC3000 (AvrRps4). Region I:At the site of pathogen infection, in the red dashed-line areas. Region II: Regions adjacent to the site of infection, in the yellow dashed-line areas and outside the red dashed-line areas. Region III: Uninfected systemic tissues, outside the yellow dashed-line areas. This experiment was repeated three times with similar results. B. False color images and quantitative analyses of the changes of chlorophyll fluorescence parameters Fv/Fm (Region 1, 2 and 3) induced by avirulent *Pst* DC3000 (AvrRps4) at 1, 2, 4 and 6 dpi, and the false color code depicted at the bottom of each image ranged from 0.000 (black) to 1.000 (purple). The statistical data of Fv/Fm support the results seen in the images.

Infection of the avirulent *Pst* DC3000 (*AvrRps4*) into *Arabidopsis* leaves lead to the spread of chlorotic disease symptoms. Additionally it caused a strong HR that becomes macroscopically apparent on day 1 post-infection (pi) at region I (the site of pathogen infection) (Figure 1A). Changes in Fv/Fm were already detectable after 3 hpi at region I. Twenty-four hpi there was also a remarkable decrease in Fv/Fm at the site of the MgCl_2_ treatment (data not shown) consistent with previous studies [20,21]. The meaning of Fv/Fm is maximum fluorescence yield of photosystem II (PS II). Several researches have shown that presence of the effectors affects ROS sources such as PS II of the chloroplast, in turn inducing the change of Fv/Fm associated with pathogen infection. It can be preliminarily inferred that at region I R proteins *RPS4* can detect the presence of pathogen effectors *AvrRps4* and quickly trigger highly diffusible downstream signaling elements like ROS and NO, which are required for the execution of HR-PCD (Hypersensitive Response-Programmed Cell Death). The primary sources of ROS include chloroplast and membrane-associated NADPH oxidase. The HR–PCD cell death was rapidly elicited within hours after pathogen attack at region I. The region II (adjacent to the site of infection) initiated an RPS4-dependent HR 2 dpi after local infection that was macroscopically apparent by 3 dpi (Figure 1A). This site usually did not experience PCD, but immediately perceived the “pro-death” ROS signals that further induced HR–PCD to kill pathogens and limit spread to adjacent tissues. It can be seen that region III had no HR in response to *Pst* DC3000 (*AvrRps4*), but Fv/Fm in Point 1, 2, and 3 significantly decreased by 19.4%, 25% and 18.8% at 6 dpi compared with 1 dpi parameters, respectively (Figure 1B). It is possible that region III eliminates pro-death signals emanating from region I or integrates them into region II cells to avoid the induction of PCD to build a defense border between dying and surviving regions. Fv/Fm decreased in region III, probably owing to chloroplast degradation for needed mobilization of nitrogen or activation of defensive signaling molecules.

Infiltration of *Pst* DC3000 (*AvrRps4*) into *atg5* mutant leaves led to slightly more widespread chlorotic cell death at region II than WT (data not shown). We found that the phenotype was clearer in the older leaves between wild-type and *atg5* induced by avirulent *Pst* DC3000 (*AvrRpm1*) as reported previously [22]. In previous studies, chloroplasts participate in not only the plant resistance response, but also serve as targets of pathogens. Autophagy is involved in the defense response, which most likely includes chloroplast-mediated plant defense responses. Therefore, in the older leaves of *atg5* mutant infection with *Pst* DC3000 (*AvrRps4*) leads to chlorotic cell death spreading to all the leaves. Because the plant was autophagy-deficient and experienced senescence, autophagy-mediated chloroplastic defense responses became inoperative, leading to chlorotic cell death spreading to all the leaves. In older leaves of wild-type or normal leaves of *atg5-1*, autophagy or chloroplasts may involve less efficient defense responses. We preliminarily consider autophagy-mediated chloroplastic defense responses most likely initiate HR–PCD to inhibit pathogens and reduce infection to adjacent tissues.

### Pst DC3000 (AvrRps4) Infection Induces GFP Bodies in Living Cells using Stroma-Targeted GFP (CT-GFP)

**Figure 2:**
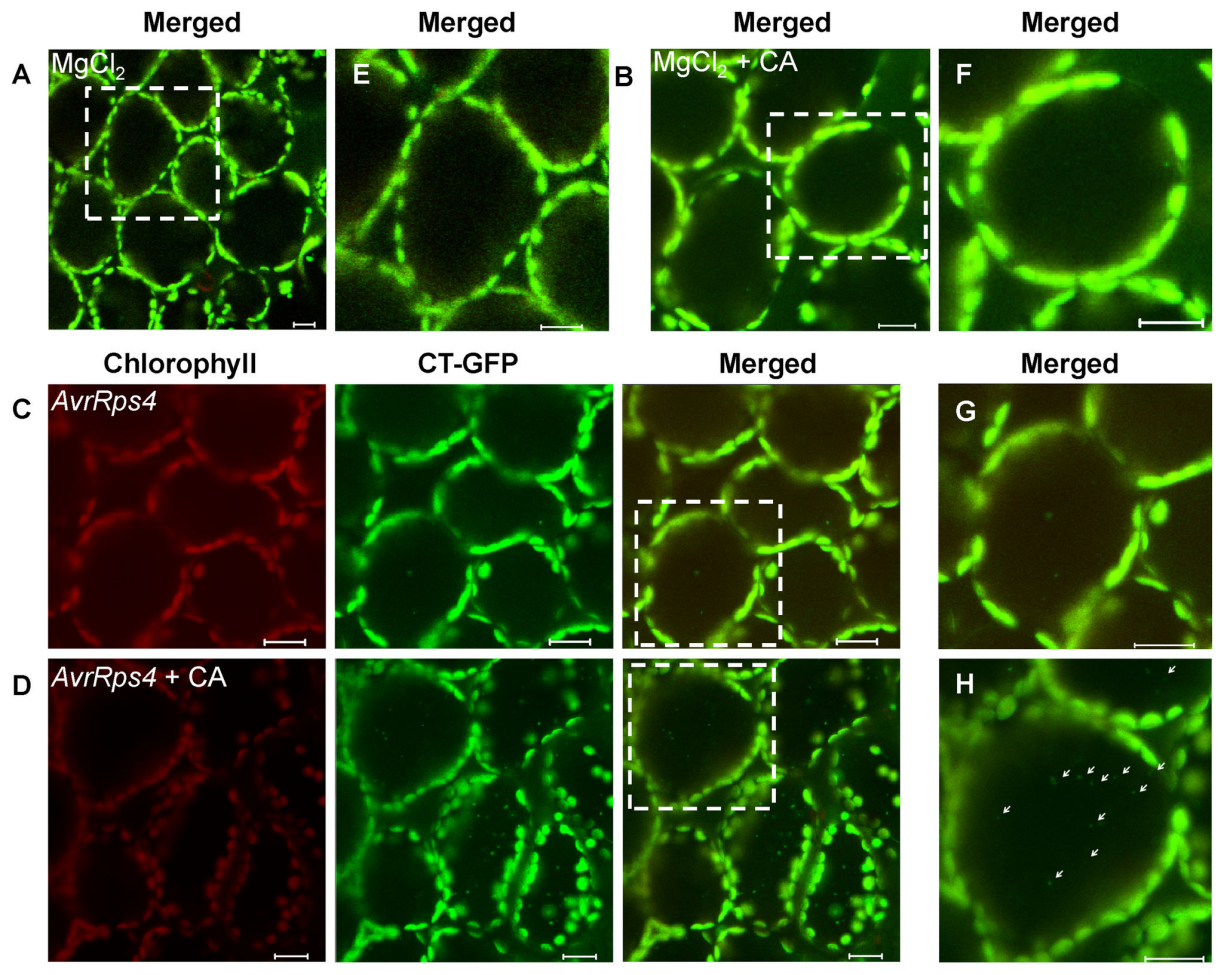
Stroma-targeted GFP bodies induction upon avirulent *Pst* DC3000 (*AvrRps4*) infection in concanamycin A-treated leaves. A, B and C. Mesophyll cells of fresh leaves excised from the plant were infiltrated with 10 mM MgCl_2_ (A) or avirulent *Pst* DC3000 (AvrRps4) (OD_600_ = 0.1) (B and C) and incubated in 10 mM MES-NaOH (pH 5.5) (A and B) or in 10 mM MES-NaOH (pH 5.5) with the addition of 1 µM CA (C) at 23 °C for 12 h. D, E and F, Magnification of a mesophyll cell of leaves incubated in the conditions described for A, B and C, respectively. Chlorophyll fluorescence appears red, and CT-GFP (Stroma-targeted GFP) appears green. In merged images, the overlap of GFP and chlorophyll fluorescence appears yellow. Spherical bodies only having GFP (arrowheads) were observed. Scale bars represent 20 μm.

We used transgenic *Arabidopsis* Stroma-Targeted GFP (CT-GFP) as experimental material as previously described [23,24]. The CT-GFP construct was fused to a double 35S promoter, the *Arabidopsis* *recA* transit peptide (CT) [25] and S65TmGFP4. CT-GFP was targeted to the stroma of chloroplasts. GFP fluorescence was observed in chloroplasts but not in the vacuole of the mesophyll cells when leaves were excised from the plant and infiltrated with 10 mM MgCl_2_ (Figure 2A, E) as previously reported [9]. This phenomenon was also observed in leaves infiltrated with 10 mM MgCl_2_ (control) and incubated in 10 mM MES-NaOH (pH 5.5) with concanamycin A (1 μM) following irradiation, or in Suc-containing MS medium in darkness (Figure 2B, F); or infected with *Pst* DC3000 (*AvrRps4*) and incubated in 10 mM MES-NaOH (pH 5.5) (Figure 2C, G), although few GFP-degradative bodies were detected. Concanamycin A (CA) is a V-ATPase inhibitor that raises the interior pH of the vacuole, and commonly blocks vacuolar lytic activity and the accumulation of degradable bodies in the vacuole [26–28]. However, more GFP bodies were detected in vacuoles of excised fresh leaves incubated in 10 mM MES-NaOH (pH 5.5) with the addition of 1 μM CA at 23 °C after avirulent *Pst* DC3000 (*AvrRps4*) infection than control (Figure 2D, H). GFP bodies exhibited random motion in cells (Figure S1B). In protoplasts, under the same treatment conditions, GFP bodies were observed moving randomly towards the center of the protoplast (Figure S1A). Taken together, these observations indicate that plants with *Pst* DC3000 (*AvrRps4*) infection may induce chloroplast degradation, which can be detected by GFP bodies, and suggest an R gene-mediated defense response.

### Pst DC3000 (AvrRps4) Infection Induces Chloroplast-Degradation GFP Bodies to the Vacuole for Autophagy

**Figure 3:**
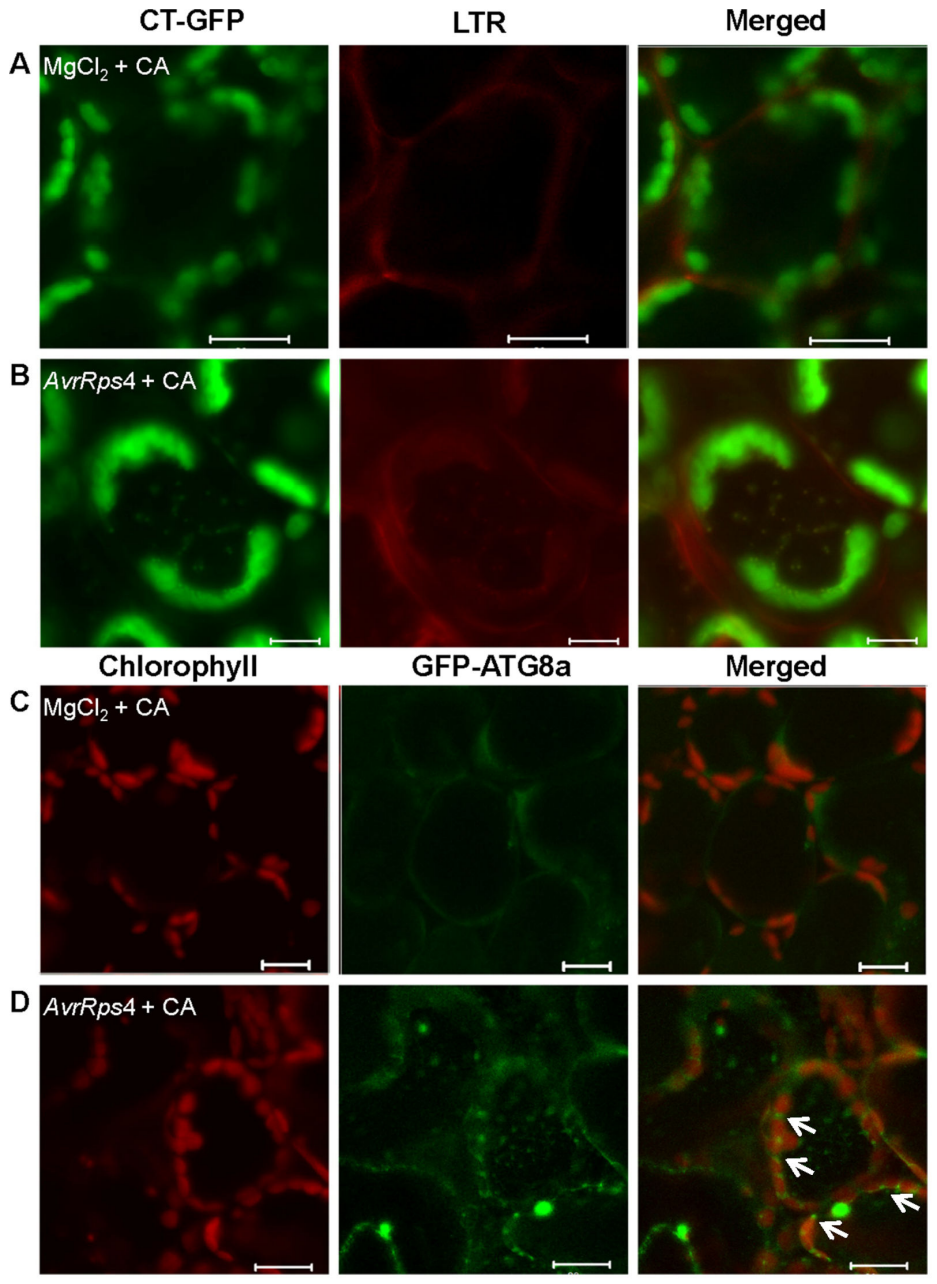
Autophagy induction upon avirulent *Pst* DC3000 (*AvrRps4*) infection in concanamycin A-treated leaves. A and B. Mesophyll cells of fresh leaves excised from the *Arabidopsis* expressing CT-GFP protein were infiltrated with 10 mM MgCl_2_ (A) or avirulent *Pst* DC3000 (AvrRps4) (B) and incubated in 10 mM MES-NaOH (pH 5.5) with the addition of 1 μM CA at 23 °C for 12 h, leaves were vacuum-infiltrated with 1 μM LTR and kept for an additional hour. C and D. Mesophyll cells of fresh leaves excised from the *Arabidopsis* expressing the GFP-ATG8a fusion protein were infiltrated with 10 mM MgCl_2_ (C) or avirulent *Pst* DC3000 (AvrRps4) (D) and incubated in 10 mM MES-NaOH (pH 5.5) with the addition of 1 μM CA at 23 °C for 15 h. A and B. LTR staining of autophagosomal-like structures appears red, and CT-GFP (Stroma-targeted GFP) appears green. In merged images, the overlap of GFP and LTR staining of autophagosomal-like structures appears yellow. C and D. Chlorophyll fluorescence appears red, and autophagic bodies with GFP-ATG8a fusion protein appears green. Scale bars represent 20 μm.

Based on previous studies, autophagy is a self-degradation response induced in plants by different pathogens. To further understand this process, we used LysoTracker Red (LTR) dye to examine the accumulation of chloroplast-degradative GFP bodies to determine whether autophagy is associated with chloroplast degradation. LTR dye, a label for acidic organelles detection such as autophagolysosomes in live tissues, was used as an indicator of possible autophagy activity [29–31]. We rarely observed the accumulation of LysoTracker-stained autolysosome-like structures in mesophyll cells before infection or after infiltration with 10 mM MgCl_2_ (control) and incubation with CA (Figure 3A). In contrast, when the leaves were infected with *Pst* DC3000 (*AvrRps4*) and incubated under the same condition, we detected a significant increase of punctate autolysosomes in the cells. Most of the punctuations were also labeled with CT-GFP (Figure 3B). This indicated that chloroplast-degradative GFP bodies are autolysosome- like structures and autophagy may well play a role in chloroplast degradation induced by *Pst* DC3000 (*AvrRps4*).

We used transgenic *Arabidopsis* expressing a GFP-ATG8 fusion protein, which is regarded as an autophagosome marker, which accumulates spherical bodies in the vacuole [32–34] to monitor autophagy. Following treatment with *Pst* DC3000 (*AvrRps4*) and incubating with CA for 12 h, a significant number of autophagosomes labeled with GFP-ATG8a were observed in mesophyll cells (Figure 3D), whereas only a few diffuse-staining bodies were detected in 10 mM MgCl_2_ treated cells (Figure 3C); some GFP-ATG8a bodies were observed at the end of oval shaped chloroplast (Figure 3D, arrowheads). We propose that it is an incipient characteristic of autophagy that plays a role in chloroplast degradation by membrane isolation and induction of generating autophagosome. However, we found that the number of autophagosomes labeled with GFP-ATG8a (Figure 3D) was relatively higher than chloroplast-degradative bodies labeled with CT-GFP (Figure 2D, H). Simultaneously, we observed that most autophagic bodies (LysoTracker-stained) overlapped with CT-GFP in punctate bodies of the vacuole; however, some were not labeled with CT-GFP (Figure S2, arrowheads). We suggest that autophagy not only induces chloroplast degradation, which involves the R gene-mediated defense response, but also induces other defense responses such as innate immunity.

**Figure 4:**
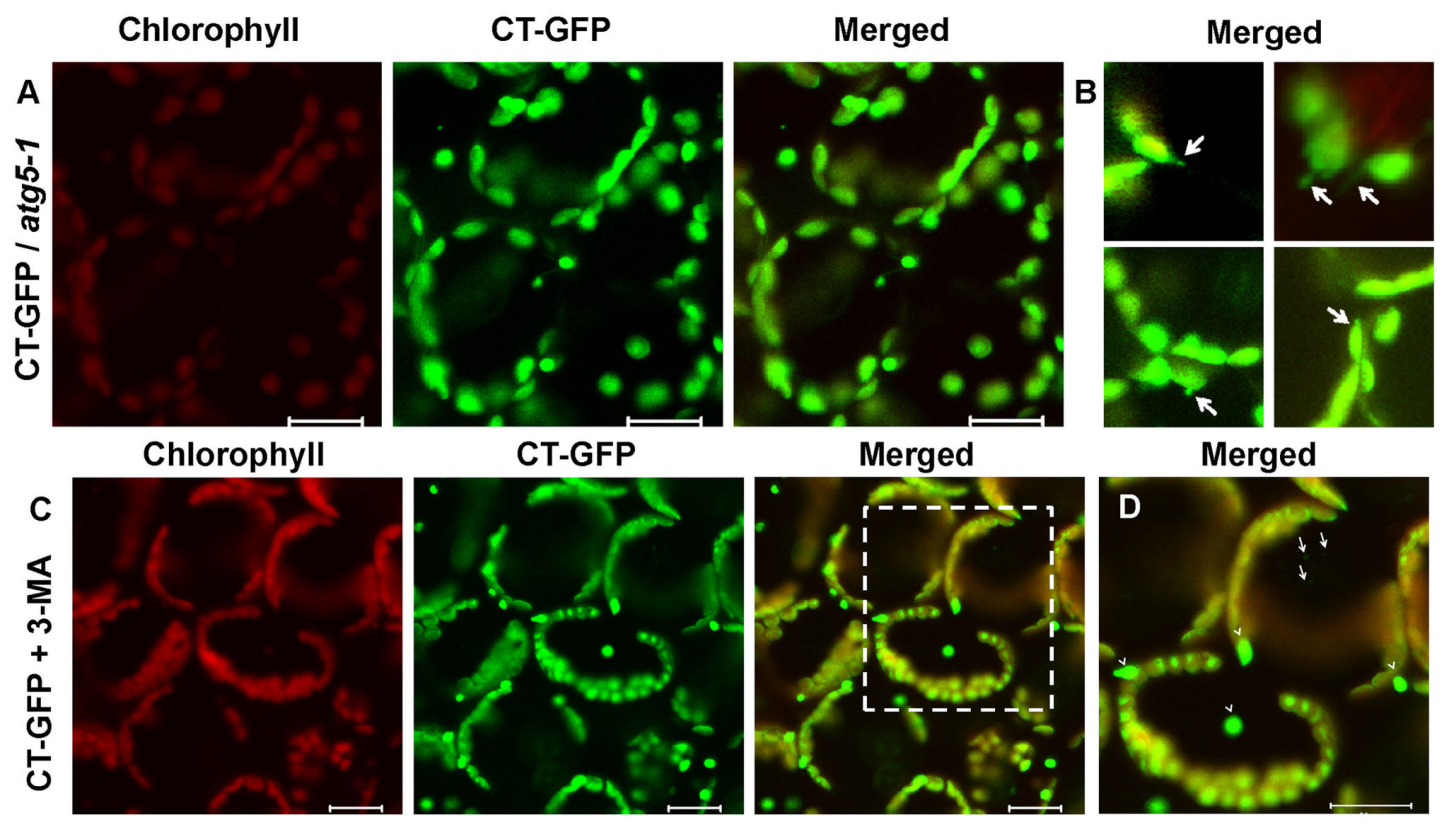
Effect of autophagic deficiency on the behavior of chloroplast degradation in mesophyll cells. A, B, C and D. Mesophyll cells of fresh leaves excised from the CT-GFP transgenic *atg5-1* plant (A and B) or CT-GFP plant (C and D) were infiltrated with 10 mM MgCl_2_ (A) or avirulent *Pst* DC3000 (AvrRps4) (OD_600_ = 0.1) (B, C and D) and incubated in 10 mM MES-NaOH (pH 5.5) with the addition of 1 μM CA (A and B) or in 10 mM MES-NaOH (pH 5.5) with the addition of 1 μM CA and 10 μM 3-MA (C and D) at 23 °C for 12 h. D, Magnification of a mesophyll cell of leaves incubated in the conditions described for C, respectively. Chlorophyll fluorescence appears red, and CT-GFP appears green. In merged images, the overlap of GFP and chlorophyll fluorescence appears yellow. Spherical bodies only having GFP (arrows) and whole chloroplast degradative bodies (arrowheads) were observed. Scale bars represent 20 μm.

To confirm whether autophagy can induce chloroplast degradation and lead to the accumulation of GFP degradative bodies, we introduced the CT-GFP into *atg5-1* mutant backgrounds by crossing and identifying homozygous *atg5-1* seedlings expressing the CT-GFP transgene by basta and kanamycin dual-resistance, and verifying by LSCM microscopy. Previous studies have revealed that *atg5-1* plants do not form ATG12-ATG5 conjugates. Because the conjugate is essential for autophagy, disruption of the ATG12-ATG5 conjugation pathway effectively abrogates the ATG8 and ATG12 conjugation pathways simultaneously in plant cells [9,32]. Our laboratory initially confirmed that ATG5 is required for limiting HR–PCD at an initial stage when induced by *Pseudomonas syringae* through SA signaling in *Arabidopsis* . Accumulation of GFP bodies in the vacuoles could not be observed when the leaves were excised from CT-GFP transgenic *atg5-1* plants during inoculation of *Pst* DC3000 (*AvrRps4*) or MgCl_2_ (control), even after CA treatment (Figure 4A, B). Interestingly, instead of the GFP signal diffusion in the cytoplasm (control, Figure 4A), there were short stroma-filled tubules labeled with CT-GFP that formed on the surface of chloroplasts (Figure 4B, Figure S4) [35,36]. We inferred that because ATG5 genes do not *a priori* affect ATG8 conjugation, autophagy plays an incipient role by the ATG8 conjugation pathway in chloroplast degradation, but disruption of ATG12-ATG5 conjugation pathway abrogates the formation of autophagy-mediated chloroplast-degradative bodies. 3-methyladenine (3-MA) not only blocks the formation of autophagosomes, but also inhibits protein degradation in cells efficiently, without affecting cellular activities, such as protein synthesis, simultaneously [37,38]; therefore, 3-MA is a very efficient inhibitor of autophagy. Leaves were excised from the CT-GFP transgenic plants and incubated in 10 mM MES-NaOH (pH 5.5) containing CA and 3-MA after *Pst* DC3000 (*AvrRps4*) infection. In the leaf cells, we can observed few chloroplast-degradative bodies (Figure 4C, D, arrowhead) and some whole degraded chloroplasts labeled with CT-GFP but without chlorophyll fluorescence in the vacuole (Figure 4D, the dashed-line areas), suggesting that the presence of both CA and 3-MA does not induce the accumulation of GFP degradative bodies but induces whole chloroplast degradation. Whole chloroplast degradation during senescence represents a suboptimal system, as previously described [11]. These results preliminarily support the assertion that chloroplast-degradative GFP bodies induced by *Pst* DC3000 (*AvrRps4*) to the vacuole is mediated mostly by autophagy. Doelling et al. (2002) found that the speed of chloroplast protein degradation is more accelerated in the *atg* mutants than in wild-type plants [19,39]. Wide spread chlorotic characteristic were observed in *atg5* mutants induced by *Pst* DC3000 (*AvrRps4*). Research by Hofius et al. (2009) found that *atg* mutants treated with cathepsin inhibitors were suppressed in HR cell death induced by avirulent pathogen. Therefore, it is likely that chloroplast degradation is mediated by chloroplast-specific or other systems of degradation such as protease cathepsin B, in addition to autophagy. Furthermore, autophagy may be the initial process for the degradation of chloroplast proteins.

**Figure 5:**
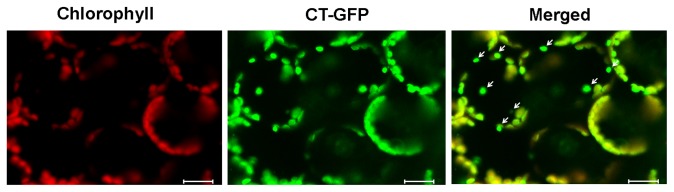
Chloroplast degradation induction upon virulent *Pst* DC3000 infection in concanamycin A-treated leaves. Mesophyll cells of fresh leaves excised from the plant were infected with virulent *Pst* DC3000 (OD_600_ = 0.1) and incubated in 10 mM MES-NaOH (pH 5.5) with the addition of 1 μM CA (C) at 23 °C for 12 h. Respectively, chlorophyll fluorescence appears red, and CT-GFP (Stroma-targeted GFP) appears green. In merged images, the overlap of GFP and chlorophyll fluorescence appears yellow. Whole chloroplast degradative bodies (arrowheads) were observed. Scale bars represent 20 μm.

A recent study by Hofius et al. (2009) supports the hypothesis that activation of TIR-NB-LRR immune receptor RPS4-mediated immune responses induces autophagy during *Pst* DC3000 (*AvrRps4*) infection. We preliminarily speculated that RPS4- mediated immune responses appear to be required for induction of chloroplast degradation via autophagy. We used the virulent *Pst* DC3000, which does not lead to a R gene-mediated defense, to infect the CT-GFP transgenic plant. After incubation in CA, the plant responded in a similar manner to the 3-MA treated plant cells which was avirulent *Pst* DC3000 (*AvrRps4*) infected. We observed few GFP degradative bodies and some whole chloroplast degradation (Figure 5). According to the results, we can infer that RPS4 appears to be required for induction of chloroplast degradation via autophagy.

### Chloroplast Degradation via Autophagy Enhances Resistance Responses of Plants to Pathogens

Chloroplasts are central to plant metabolism, such as photosynthesis for the assimilation of nutrients and synthesis of various metabolites such as hormones. Increasing evidence suggests that the chloroplast exerts an important function during pathogen infection. The chloroplast is the primary source of ROS, SA, and JA in the plant cell [40]. Caplan et al. (2008) confirmed that the chloroplastic protein NRIP1 mediates innate N immune receptor recognition of a viral effector (p50, the 50 kDa helicase domain of TMV’s replicase) [41]. The N immune receptor and RPS4 receptor belong to the TIR-NB-LRR class. Mühlenbock et al. (2008) also reported that chloroplast signaling regulates the crosstalk between light acclimation and immunity with *lsd1* in *Arabidopsis* [42]. The chloroplast induces unchecked ROS production to suppress pathogens when the plant is exposed to excess light and continuous photoperiods. The ultrastructure of the chloroplasts under low light is significantly changed, in number and area. In normal light, the mesophyll cells of leaves have more chloroplasts than the corresponding cells of low light leaves [43]. Therefore, we used wild-type and *atg5-1* mutant plants grown in a plant growth chamber with normal light and low light period for 3 weeks. The number of chloroplasts in normal light cells was approximately 10%-15% more than low light cells (Figure S3A, B).

**Figure 6:**
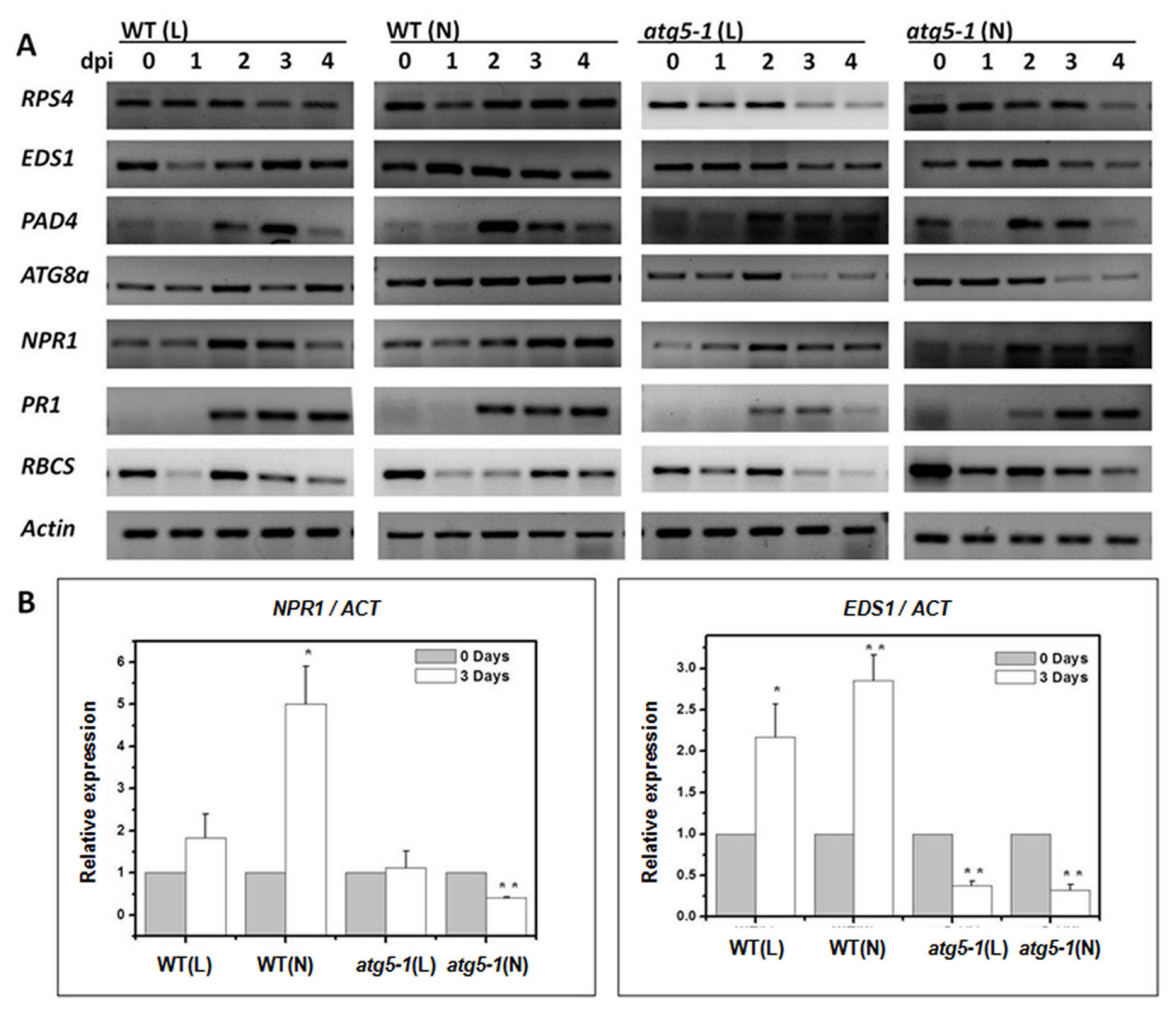
Expression pattern of related genes in wild-type (WT) and *atg5-1* plants. A. Expression of *RPS4, EDS1, PAD4, ATG8a, NPR1, PR1* and *RBCS* in normal light (N) environment and low light (L) environment of wild-type and *atg5-1* plants during the avirulent *Pst* DC3000 (AvrRps4) treatment. Total RNA was isolated from third and fourth leaves (about 0.1g, collected at 0, 1, 2, 3 and 4 days) of each plant and subjected to semiquantitative RT-PCR using gene-speciﬁc primers. 18s ribosomal RNA was used as an internal control. B. Q-PCR quantiﬁcation of *NPR1* and *EDS1* mRNA levels in WT (gray), *atg5-1* (white) 0 and 2 or 3 d after inoculation with avirulent *Pst* DC3000 (AvrRps4). Error bars represent SD of the mean and standard deviation of values obtained from three biological samples per genotype and time point. The asterisk indicates a signiﬁcant difference from control (*, *P* < 0.05; **, *P*<0.01).

Based on these differences, we examined gene expression to confirm the role of chloroplast degradation via autophagy during *Pst* DC3000 (*AvrRps4*) infection. *RPS4* belongs to the TIR-NBS-LRR class of *R* genes in *Arabidopsis* and requires *EDS1* and *PAD4* to confer resistance [44–47]. Both the wild-type and *atg5-1* plants showed rapid accumulation of *RPS4* and *EDS1* transcripts after inoculation, and they were maintained at high levels in wild-type plants, especially in normal light. In contrast, *RPS4* and *EDS1* transcripts in *atg5-1* reached a peak at 3 dpi and decreased at 3-4 dpi (Figure 6A). This suggests that *RPS4* and *EDS1* are essential components of effector-triggered immunity (ETI) and recognize specific pathogen effectors and act upstream of autophagy. Chloroplast degradation via autophagy in late stages may play a role in maintaining the level of *RPS4* and EDS 1 The expression pattern of *PAD4* both in the wild-type and *atg5-1* was low at the initial stage, but reached a peak at 2 dpi. Furthermore, the level in normal light wild-type was higher than that of the wild-type (L), *atg5-1* (N) and *atg5-1* (L) (Figure 6A). This observation is similar to Rietz et al. (2011) that showed RPS4 and EDS1 first recognize *AvrRps4* then *EDS1* in combination with *PAD4* to up-regulate the transcription of *PAD4* itself and mobilize SA defenses to reinforce resistance [45–47]. The expression level of *ATG8a* increased gradually and was higher in wild-type (N) than wild-type (L), but its level decreased at 2-3 dpi in *atg5-1* (Figure 6A). We also detected that the *NPR1* transcript levels of the SA signal pathway accumulated as well as the innate immune response gene *PR1*. The expression level of *PR1* was similar in the wild-type and *atg5-1* after infection (Figure 6A). This suggests that the autophagic mutant has the capacity to induce innate defense responses [4]. The transcript accumulation of *NPR1* was higher in wild-type, especially in normal light than in the *atg5-1*. This demonstrates that chloroplast degradation via autophagy promotes the SA signal pathway mediated defense reaction.

To further confirm this result, we used real-time PCR to investigate the expression of *EDS1* and *NPR1*. Relative expressions were evidently higher in normal light wild-type than low light wild-type and *atg5-1* at 3 dpi (Figure 6A, B). These cumulative results revealed that chloroplast degradation via autophagy requires RPS4-EDS1 mediated immune defense for induction. In contrast, chloroplast degradation via autophagy promotes the immune defense and SA signal pathway mediated defense reaction at the later stage.

**Figure 7:**
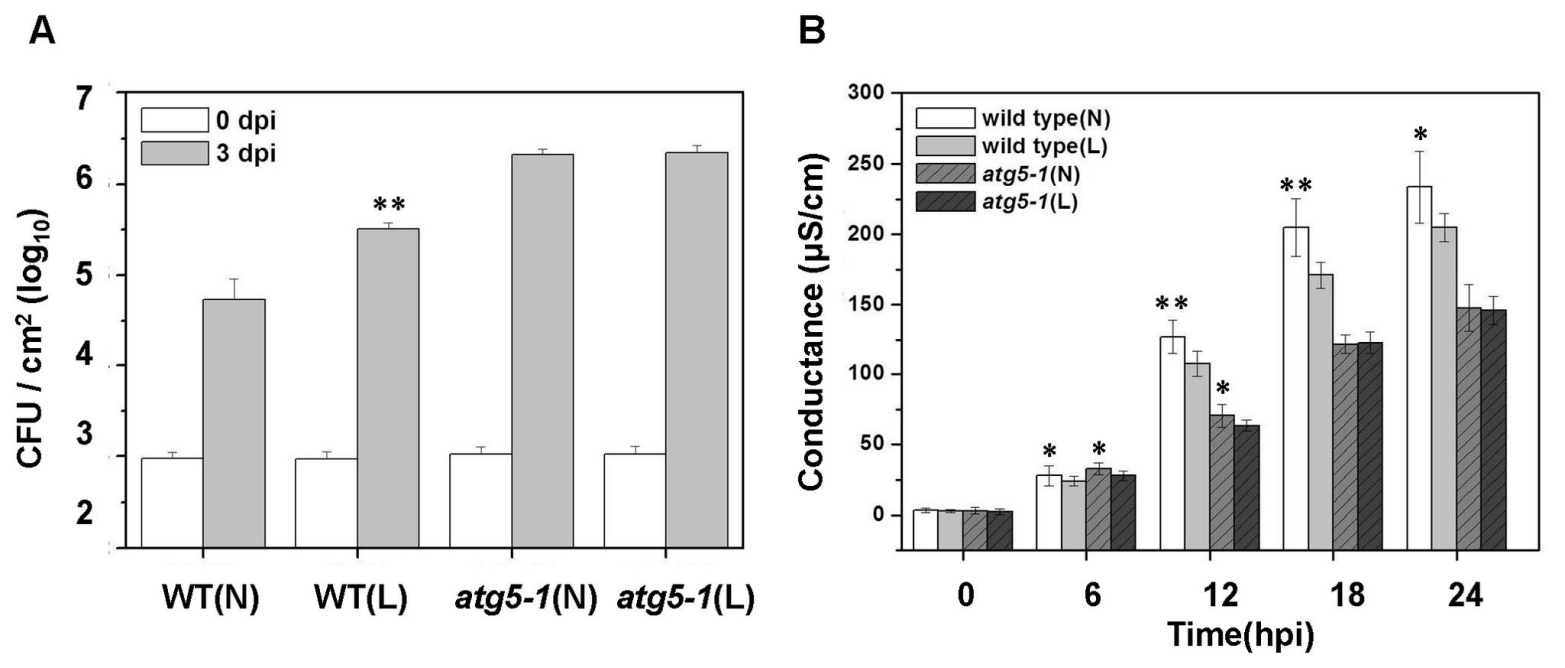
Contribution of Chloroplast via Autophagy to Disease Resistance against Avirulent *Pst* DC3000 (*AvrRps4*). A. Bacterial growth quantification of *Pst* DC3000 (AvrRps4) on wild-type and *atg5-1*, which grow in normal light (N) and low light (L) environment. 4-week-old plants were infiltrated with 1×10^5^ cfu/ml^-1^ (OD_600_ = 0.0001) and the samples were collected at 0 (white bars) and 3 dpi (gray bars) for assay. Error bars represent SD of the mean of three samples. B. Enhanced electrolyte leakage in the wild-type and *atg5-1* mutant, which grow in normal light (N) and low light (L) environment, following inoculation with avirulent *Pst* DC3000 (AvrRps4). The error bars display standard deviation (SD) from four technical replicates from two independent replicates.

In addition, we measured the effect of chloroplast degradation via autophagy on RPS4-dependent suppression of bacterial growth [48,49]. The disease susceptibility of wild-type and *atg5-1* mutant, which were grown in normal light and low light, was examined (Figure 7A). The *Pst* DC3000 (*AvrRps4*) increased to high levels by 3 days after infection. Growth of *Pst* DC3000 (*AvrRps4*) in wild-type was significantly reduced relative to *atg5-1* plants, whether the plant was grown in normal light or low light. Growth of bacteria in wild-type (L) was 3-fold higher than wild-type (N) (Figure 7A). In contrast, leaves of the *atg5-1* mutant whether grown in normal or low light permitted similar growth of *Pst* DC3000 (*AvrRps4*). To further confirm this result, we used the electrolyte leakage assay [4,50,51] to quantify the effect of *Pst* DC3000 (*AvrRps4*) induced HR cell death (Figure 7B). The difference in measured electrolytes between wild-type and *atg5-1* were greatest after 12 hpi (Figure 7B). The assays also showed a significant increase in conductance in wild-type (N) by 12 hpi (Figure 7B). However, this increase was relatively suppressed in wild-type (L) (Figure 7B). Specifically, we did not detect a significant difference in electrolyte leakage between *atg5-1*(N) and *atg5-1*(L) (Figure 7B). This was supported by similar disease susceptibility of wild-type (N and L) and *atg5-1* (N and L) infected with *Pst* DC3000 (*AvrRps4*) (Figure 7A). Thus, it appears that chloroplast degradation via autophagy enhances RPS4-dependent resistance response to *Pst* DC3000 (*AvrRps4*).

**Figure 8:**
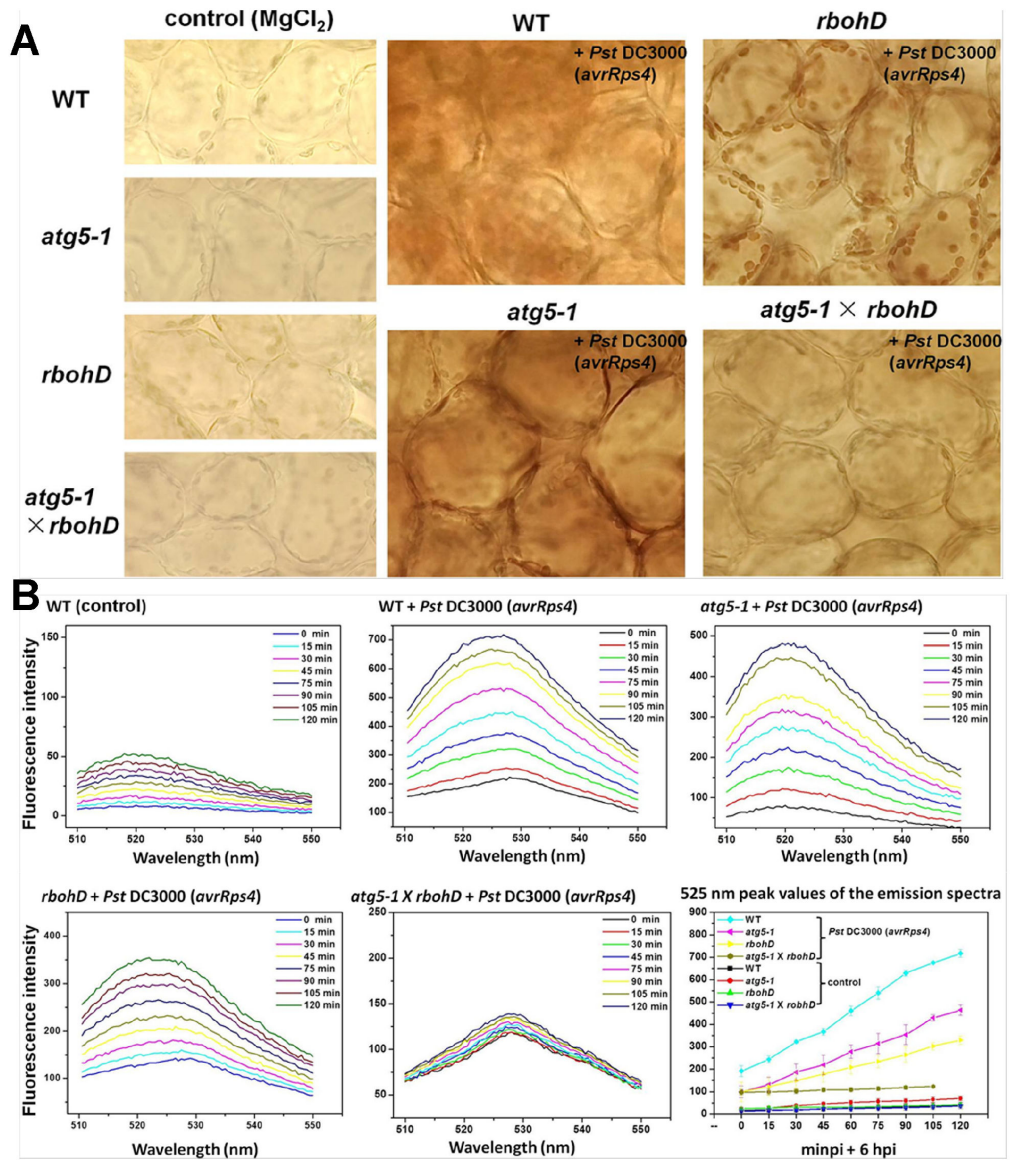
The accumulations of H_2_O_2_ induced by pathogens in WT*, atg5-1*, *rbohD* and *atg5-1* × *rbohD*. A. The plants (4 weeks old) were infiltrated with *Pst* DC3000 (AvrRps4) (OD_600_ = 0.2) or MgCl_2_ (control). DAB staining of leaves from WT, *atg5-1*, *rbohD* and *atg5-1* × *rbohD* were taken after 24 hpi, respectively. Experiments were performed three times with similar results. B. Show are *Arabidopsis* leaves after infiltrating *Pst* DC3000 (AvrRps4) (OD_600_ = 0.2) or 10 mM MgCl_2_ (control) for 6 hpi. Then the changes of 525 nm peak values in fluorescence emission spectra were scanned for 120 min. Excitation wavelength: 488 nm; Excitation slit width: 10 nm; Emission slit width: 8.5 nm; Scanning speed: 200 nm/min; Scanning wavelength range: 510-550 nm.

ROS can be detected by H_2_DCFDA (2′, 7′-dichlorofluorescin diacetate) and DAB (3,3′-diaminobenzidine) methods (Figure 8). The DAB staining results showed accumulation of H_2_O_2_ in the mesophyll cells by 24 hpi (Figure 8A). The accumulation of H_2_O_2_ in the leaves of WT infected with *Pst* DC3000 (*AvrRps4*) was stronger than other samples. In the *rbohD* mutant ( *Arabidopsis* NADPH oxidases knockout mutant), we observed that ROS only accumulated in chloroplasts of the infected leaves, however, accumulation of H_2_O_2_ in *atg5-1* mutant was weaker than WT and not clearly observed in chloroplasts. The phenomenon of H_2_O_2_ bursts was abolished in *atg5-1* × *rbohD* double mutant (Figure 8A). We used fluorescence phosphorescence spectrophotometer to detect ROS bursts in the leaves (Figure 8B). In WT, the accumulation of 525 nm peak values in fluorescence emission spectra reached approximately 700 units fluorescence intensity by 8 hpi (6 hpi + 120 min), however, the values of *rbohD* and *atg5-1*only reached 350-500 units and *atg5-1* × *rbohD* double mutant was lowest at about 100 units; the combination value of *atg5-1* with *rbohD*, the units were higher than the WT value (Figure 8B). According to this result, we suggest that autophagy may mediate chloroplast degradation and assist in inducing ROS, but chloroplasts have alternatives processes to induce ROS accumulation when autophagy is absence. These results imply that autophagy dependent chloroplast degradation may be the primary source of ROS to induce defense response.

## Discussion

### Role of Autophagy in Pst DC3000 (AvrRps4)-Induced Chloroplast Degradation

In our study, we provide direct evidence of CT-GFP bodies during *Pst* DC3000 (*AvrRps4*) infection, using live cell imaging (Figure 2D, H). We detected the accumulation of CT-GFP bodies in the vacuole only when the vacuolar lytic activity was suppressed by the addition of CA (Figure 2). This suggests that the stroma-targeted GFP bodies are probably degraded from the chloroplasts. Ishida et al. (2008) also visualized that the chloroplast-degradative CT-GFP bodies accumulated in the vacuole during senescence [9]. In addition, the mobilization of chloroplast degradative bodies by the autophagy-mediated system to the vacuole is supported by the detection of LTR staining punctate structures, a marker for the indication of autophagy activity [30,52], that colocalized with CT-GFP in the autolysosome-like body (Figure 3C). We also observed a small number of LTR staining punctuate bodies that did not colocalize with CT-GFP during *Pst* DC3000 (*AvrRps4*) infection (Figure S2). In addition, GFP-ATG8a, a marker for autophagic bodies, was visualized in punctate bodies in the vacuole (Figure 3D), and the number of punctate bodies was larger than the number of the chloroplast-degradative CT-GFP bodies (Figure 2D, H; Figure 3D). It is therefore possible that autophagy induces chloroplast degradation through mobilization to the vacuole during *Pst* DC3000 (*AvrRps4*) incubation.

A previous study by Hofius et al. (2009) demonstrated that *Pst* DC3000 (*AvrRps4*) infection the TIR-NB-LRR immune receptor RPS4 recognizes the avirulence factor *AvrRps4* and activates EDS1 to induce autophagy, which further enhances the defense response [4,45,53]. In addition, chloroplasts are not only central to photosynthesis, but also plant metabolism. There is increasing evidence to suggest that chloroplasts play a significant role during ETI [41,42,54], which may be the source of ROS and the pathogen-response signaling molecule SA. These results indicate that through RPS4-mediated immune defense response, autophagy induces the degradation of chloroplasts, which has a major role in the immune response.

According to the above inference that autophagy induces chloroplasts to be removed, and *atg5-1* mutant is defective in autophagy, it is possible that the *atg5-1* mutant decreases the rate of chloroplast degradation and chlorosis. However, in the *atg5-1* mutants, chlorotic cell death is slightly more widespread than the wild-type, and few chloroplast degradative bodies are detected in cells (Figure 4B). When treated with the autophagy inhibitor 3-MA after pathogen infection, only a few chloroplast degradative bodies and some whole degradative chloroplasts with CT-GFP without chlorophyll can be observed (Figure 4C, D). A more likely scenario is that there is not a single process for the degradation of chloroplasts during *Pst* DC3000 (*AvrRps4*) infection. Autophagy plays the initial role in chloroplast degradation, but other pathways of chloroplast degradation such as chloroplast proteases upregulation, may be involved when autophagy is impaired.

When the leaves of CT-GFP transgenic plant were infected with virulent *Pst* DC3000, few chloroplast degradative bodies are observed, even in those incubated with concanamycin A (Figure 5). Few whole degradative chloroplasts are observed in cells. We propose that RPS4-mediated immune responses appear to be required for induction of chloroplast degradation via autophagy. Whole degradative chloroplasts are not observed during avirulent *Pst* DC3000 (*AvrRps4*) infection. It is possible that autophagy does not have the ability to remove whole chloroplasts, because the chloroplasts are too large. Autophagic bodies have a diameter of only 1.5-2.0 μm in roots and leaves [4,33].

In previous studies, the mitochondria, nucleus, and endoplasmic reticulum are partially engulfed by the vacuole (piecemeal microautophagy) [55–58]. Peroxisomes and ribosomes are entirely engulfed by autophagosomes and then transported to the vacuole in yeast (macroautophagy) [59,60]. In addition, recent related reports have revealed that the degradation of cellular components to the vacuolar is required for autophagy in plants [34,36,61,62]. We preliminarily suggest that autophagy also may plays an important role in chloroplast degradation during plant resistance responses.

### The Roles of Chloroplast Degradation via Autophagy in Plant Immune Response

Several *Pst* DC3000 effectors have chloroplast targeted signal peptides [63,64]. Additionally, many pathogen effectors target chloroplasts to dampen the release of chloroplast-derived stress signals [65]. The *Pst* DC3000 cysteine protease effector protein HopN1 interferes with photosynthesis and suppresses plant innate immune responses [66]. HopI1, a J domain virulence effector from *Pst* DC3000, localizes to chloroplasts, and induces chloroplast thylakoid structure remodeling and suppresses plant defenses such as SA accumulation [17]. Chloroplasts are one of the primary hosts of pathogens, and chloroplastic proteins are targeted by pathogen effectors. The chloroplast and chloroplast proteins not only induce ROS and the pathogen-response signaling molecules to inhibit the pathogen, but also enhance immune defenses through other pathways. The chloroplast-localized Sigma Factor-binging Protein 1 (SIB1) plays a role in pathogen-response signaling molecules-mediated defense responses [67]. The TMV viral replicase effector protein targets the chloroplast-localized NRIP1, but NRIP1 recognizes the effector and acts as the signal that promotes the N immune receptor activation and HR–PCD [68]. We hypothesize that chloroplasts or chloroplastic proteins act through chloroplast degradation via autophagy to mediate innate immune receptor recognition of the viral effector and inhibit the pathogen.

We performed experiments using wild-type and *atg5-1* mutant plants to demonstrate growth in normal light (N) and in low light (L) environments leads to different numbers of chloroplasts (Figure S3A, B). We also examined gene expression (Figure 6), suppression of bacterial growth (Figure 7A), the electrolyte leakage assay (Figure 7B) and the generation of ROS (Figure 8) to confirm the role of chloroplast degradation via autophagy during *Pst* DC3000 (*AvrRps4*) infection. There is not a great difference between *atg5-1* (N) and *atg5-1* (L) plants (Figures 6, 7). However, expression of RPS4, EDS1, and NPR1 in wild-type (N), are significantly higher than in wild-type (L) after 3 dpi (Figure 6). In contrast, growth of *Pst* DC3000 (*AvrRps4*) in wild-type (N) is lower than wild-type (L) at 3 dpi (Figure 7A). These assays show a significant increase in conductance in wild-type (N) by 12 hpi, but a slower increase in wild-type (L) than wild-type (N) (Figure 7B). Therefore, we propose that chloroplast degradation via autophagy plays a role in immune defenses.

Chloroplasts historically have been viewed as a major attack site for pathogens, particularly chloropastic proteins. Chloroplasts are not only central to photosynthesis, but also central to plant metabolism. A growing body of evidence suggests that chloroplasts are the ‘primary weapon’ for killing pathogens. Autophagy may be involved in the removal of disrupted chloroplast or chloroplastic proteins and most likely mobilize required nitrogen, trigger the accumulation of ROS and pathogen-response signaling molecules to promote immune defenses. 

## Materials and Methods

### Bacterial strains, growth and inoculation

The *P. syringae* strain (*Pst* DC3000 (*AvrRps4*), provided by Dr. Yang of South China Normal University) was cultured in King’s B medium containing rifampicin (100 µg/ml) and kanamycin (100 µg/ml) at 28 °C for 18 h. The pathogens were harvested by centrifugation (4000 rpm/min, twice), washed with 10 mM MgCl_2_ (twice), then resuspended in 10 mM MgCl_2_ and diluted to the desired density (OD_600_ = 0.2, 2-4 × 10^8^ cfu/ml) [69].

### Confocal microscopy

Before visualization, mesophyll cells of fresh leaves excised from the *Arabidopsis* expressing stroma-targeted GFP(CT-GFP) or expressing the GFP-ATG8a fusion protein were infiltrated with 10 mM MgCl_2_ (A) or avirulent *Pst* DC3000 (*AvrRps4*) (B) and incubated in 10 mM MES-NaOH (pH 5.5) with the addition of 1 µM CA at 23 °C for 12 h. The stroma-targeted GFP (CT-GFP) or the GFP-ATG8a fusion protein (GFP-ATG8a) of leaves was detected by confocal microscopy with excitation at 488 nm (a multi-Ar ion laser) and emission at 505-550 nm. Chlorophyll of leaves was excited with the 488-nm line of a multi-Ar ion laser and it was detected with emission at 650-730 nm by a multichannel detector with filters. Leaves were Syringe-infiltrated with 10 mM MgCl_2_ or avirulent *Pst* DC3000 (*AvrRps4*) and incubated in 1 μM LTR (DND-99, Invitrogen) at time points for additional 1 h after bacterial infection in darkness. LysoTracker Red (LTR) fluorescence indicative of autophagy activity was detected by confocal microscopy with excitation at 543 nm (a l mW helium: neon laser) and emission at 560-615 nm. For observation of leaves expressing the stroma-targeted GFP (CT-GFP) and LTR fluorescence, GFP was excited with the 488-nm line of a multi-Ar ion laser and LTR fluorescence was excited with the 543-nm line of a l mW helium: neon laser. A Zeiss Observer Z1 epifluorescence motorized microscope coupled to a Zeiss LSM 510 META system (LCSM, LSM510/ConfoCor2, Carl-Zeiss, Jena, Germany) was used. The system was controlled by LSM software (version 4.2). Images were obtained by the 40 × oil immersion objective and analyzed with Aim Image Browser Image Processing software (Carl Zeiss) [9,31,70].

### Measurement of the Number of Chloroplasts

The procedure followed was essentially that described by Kevin and Rachel. Entire leaf of *Arabidopsis* segment was firstly fixed in 3.5% (v/v) glutaraldehyde for 1h in the dark. The segments were then rinsed with 0.1 M Na _2_EDTA (pH 9) and the stationary liquid (3.5% (v/v) glutaraldehyde) was replaced by Na _2_EDTA. The softening leaf segment was optimal after the EDTA-treated tissue less than 1 h. The tissue was washed with distilled H_2_O had been incubated in a shaking (300 oscillations/min) water bath at 60 °C for 2.5 h. Chloroplasts in the separated mesophyll cells obtained by the maceration of prepared leaf tissue on a microscope slide were counted with a Zeiss Observer Z1 epifluorescence motorized microscope coupled to a Zeiss LSM 510 system (LCSM, LSM510/ ConfoCor2, Carl Zeiss, Jena, Germany) on differential interference contrast images [10,71].

### Bacterial pathogen counting

The four-week-old plants were vacuum infiltrated with *Pst* DC3000 (*AvrRps4*) suspended at 10^4^ cfu/ml in 10 mM MgCl_2_ and kept covered for 24 h. The infected leaves were harvested in several time points and sterilized in a 70% ethanol solution for 1 min. Leaf disks were bored from the infiltrated area and excised from leaves with a 0.5 cm^2^. Then the single sample was placed in a 1.5 ml microfuge tube with 100 µl sterile distilled H_2_O and thoroughly vortexed. The leaf disks for a single sample were placed in a 1.5 ml microfuge tube with 100 µl sterile distilled water. The pestle was rinsed with 900 µl of water, with the rinse being collected in the original sample tube and serially diluted to measure bacterial numbers until got countable colonies. 100 µl of a single sample is spread on a single plate (the King’s B medium supplemented with the necessary antibiotics). The plates are placed at 28 °C for approximately 48 h and then the colony-forming units could be counted. We counted the dilution that gave us between 1 and 20 colonies [48,72].

### Gene expression analysis

The four-week-old plants were (Col-0 and *atg5-1*) were dipping inoculated with *Pst* DC3000 (*AvrRps4*) (OD_600_ = 0.2, 1-2 × 10^8^ cfu/ml) and 10 mM MgCl_2_ [0.02 to 0.05% Silwet L-77 (S5505，GE Healthcare)]. RNA was isolated from leaves (0.1 g, collected at 0, 1, 2, 3 and 4 days and frozen in liquid nitrogen and stored at -80 °C) using TRIzol reagent (Invitrogen, Guangzhou, China). RNA concentrations were checked and the quality and accuracy of the concentration was verified with BioPhotometer plus (Eppendorf) and electrophoresis. Total RNA was treated with Reverse Transcriptase M-MLV (RNase H^-^) (Takara Bio) to synthesize the first-strand cDNA [68,73]. Gene-specific primers used for pathogen defensive marker gene PCR were 5′ -GGAAGAAGCAGGAGCAGT- 3′ and 5′-GTCACCAACCAAAGGAGC- 3′ for *EDS1*; 5′ -ATGACATCGCCGGGATTACA- 3′ and 5′ -CCAAAGTGCGGTGAAAG- C- 3′ for *PAD4*; 5′ -TTCGGCTGAAGCAATGAG- 3′ and 5′ -GTCGCGGTCTAAG- CTCGT- 3′ for *RPS4*; 5′ -CCGATAACACCGACTCCTC- 3′ and 5′ -CTTGAAGAT- GAAAGCCAAATAG- 3′ for *NPR1*; 5′ -CTCAAGATAGCCCACAAGATT -3′ and 5′ -GCGTAGTTGTAGTTAGCCTTCT- 3′ for *PR1*; Gene-specific primers used for Chloroplasts related gene PCR was 5′ -ACCTTCTCCGCAACAAGTGG- 3′ and 5′ -G- AAGCTTGGTGGCTTGTAGG- 3′ for *RBCS2B* [10,74]; Gene-specific primers used for autophagy gene PCR were 5′ -TCCCCCGGGATGATCTTTGCTTGCTTGA- 3′ and 5′ -CGGGATCCAGCAACGGTAAGAGATCCA- 3′ for *ATG8a*; 5′ -ATGGCGAA- GGAAGCGGTCA- 3′ and 5′ -CACAAAGGAGATCGAAAAGAACAC- 3′ for *ATG5* [10,32]; and the *Actin* gene (18s ribosomal RNA) was used as a control [70,75]. PCR was terminated after 28 cycles for *Actin*, *PAD4*, *RPS4*, and *NPR1*, 27 cycles for *ATG5*, *ATG8a* and *EDS1*, 20 cycles for *RCBS2B* and *PR1*. Gene-specific primers used for real-time PCR were 5′ -CAATTCATCGGAACCTGTTG- 3′ and 5′ -GAGGAGTC- GGTGTTATCGGT- 3′ for *NPR1* (103bp); 5′ -CCAATTGGATCCCAGAAAGT- 3′ and 5′ -AACAGCTTGGTTTGCAACAG- 3′ for *EDS1* (106bp). The level of relative expression was analyzed by the 2^△△Ct^ analysis method [51,71].

### Ion leakage

Ion leakage assay was performed as previously described [4,51,72], with some modifications. The leaves of 4-week-old wild-type and *atg5-1* plants were infiltrated with *Pst* DC3000 (*AvrRps4*) or MgCl_2_, and 6 leaf discs (8 mM diameter) were removed rapidly following infection and washed in 50 ml ddH_2_O (twice). After 10 min, we removed the wash water and replaced it with 15 ml of ddH_2_O. Ion leakage was then measured over time.

### H_2_O_2_ Staining, Microscopy and Scanning

Accumulation of H_2_O_2_ was visualized by staining *Arabidopsis* leaves with 3, 3’- diaminobenzidine (DAB) (D8001, Sigma) or detected with H_2_DCFDA (Molecular Probes, D6883, Sigma). The leaves (4 weeks old) were infiltrated with *Pst* DC3000 (*AvrRps4*) (OD_600_ = 0.2) or MgCl_2_ (control) at 24 hpi point and vacuum filtrated with 0.1% DAB solution for 5 min, exposed to light for 2 h. Then cleared by boiling in alcohol for 10 min and washed twice with double distilled water. The samples were stored in 50% glycerol and photographed with a Zeiss LSM 510 META microscope and digital camera.

The Infiltrated leaves at 6 hpi were incubated in 5 μM H_2_DCFDA (12.5 μl 200 μM stock solution + 487.5 μl ddH_2_O) for 15 min in darkness, and rinsed with ddH_2_O. The changes of 525 nm peak values in fluorescence emission spectra were scanned by fluorescence phosphorescence spectrophotometer (LS55, PerkinElmer, BeaconsWeld, Bucks, UK) for 120 min. Main Parameters: Excitation wavelength, 488 nm; Excitation slit width, 10 nm; Emission slit width, 8.5 nm; Scanning speed, 200 nm/min; Scanning wavelength range, 510-550 nm.

### Statistical analysis

All results were repeated at least three times and independently of each other. Statistical analysis was performed with an ANOVA with Student’s paired t test. Statistical significance was accepted at the level of * P <0.05, ** P <0.01.

### Accession numbers

Sequence data from this article can be found in The *Arabidopsis* Information Resource (http://www.Arabidopsis.org/) or Gene/NCBI databases (http://www.ncbi.nlm.nih.gov/gene/) under the following accession numbers: At3g18780.2 (*Actin*), At4g21980 (*ATG8a*), At5g17290 (*ATG5*), At1g64280 (*NPR1*), At5g45250 (*RPS4*), At3g48090 (*EDS1*), At3g52430 (*PAD4*) and At2g14610 (*PR1*). The *Arabidopsis* mutant from this article can be found in The European *Arabidopsis* Stock Centre (NASC, http://Arabidopsis.info/) under the following accession numbers: SAIL_129B07 (*atg5-1*), N9555 (*rbohD*).

## Supporting Information

Figure S1 **Movement of GFP degradative bodies in mesophyll cells or protoplasts of CT-GFP plants infected with *Pst* DC3000 (AvrRps4) and incubated in MES-NaOH(pH 5.5) with 1 µM CA for 12 h.** Protoplasts were made from the *Pst* DC3000-infected and the CA-treated leaves by the procedure of Ishida et al. (2000) [9,76] and observed by the procedure of Li and Xing (2011) [77]..
(TIF)

Figure S2 **Visualization of CT-GFP and LTR staining of autophagosomal-related structures in mesophyll cells of *Arabidopsis* by LSCM.**
(TIF)

Figure S3 **Differential interference contrast images of chloroplasts in mesophyll cells removed from leaves of wild-type and *atg5-1* plants (A) and photographs of leaves of wild-type and *atg5-1* plants (B and C).** The wild-type and *atg5-1* plants were respectively grown in a plant growth chamber with normal light and low light period for 3 weeks.
(TIFF)

Figure S4 **Visualization of the CT-GFP transgenic *atg5-1* plant structures in guard cell of *Arabidopsis* by LSCM.**
(TIF)
