# Supplementary figures and images for: The Role of Autophagy in Chloroplast Degradation and Chlorophagy in Immune Defenses during Pst DC3000 (AvrRps4) Infection
Source: PLoS One. 2013 Aug 30;8(8):e73091. doi: 10.1371/journal.pone.0073091 (PMC3758262; doi:10.1371/journal.pone.0073091)

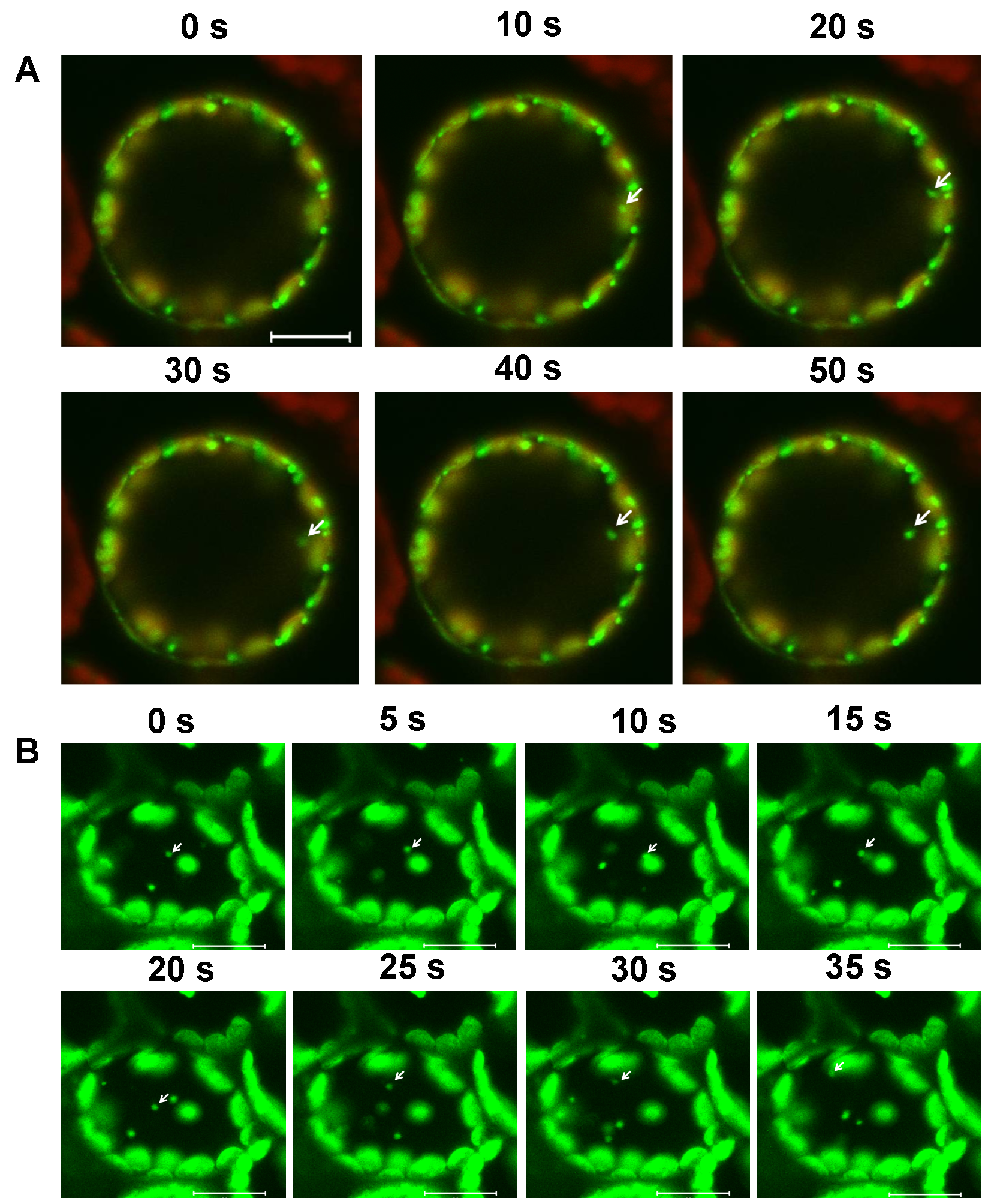

Supplement: Figure S1 — Movement of GFP degradative bodies in mesophyll cells or protoplasts of CT-GFP plants infected with Pst DC3000 (AvrRps4) and incubated in MES-NaOH(pH 5.5) with 1 µM CA for 12 h. Protoplasts were made from the Pst DC3000-infected and the CA-treated leaves by the procedure of Ishida et al. (2000) [9,76] and observed by the procedure of Li and Xing (2011) [77].. (TIF) [file pone.0073091.s001.tif]

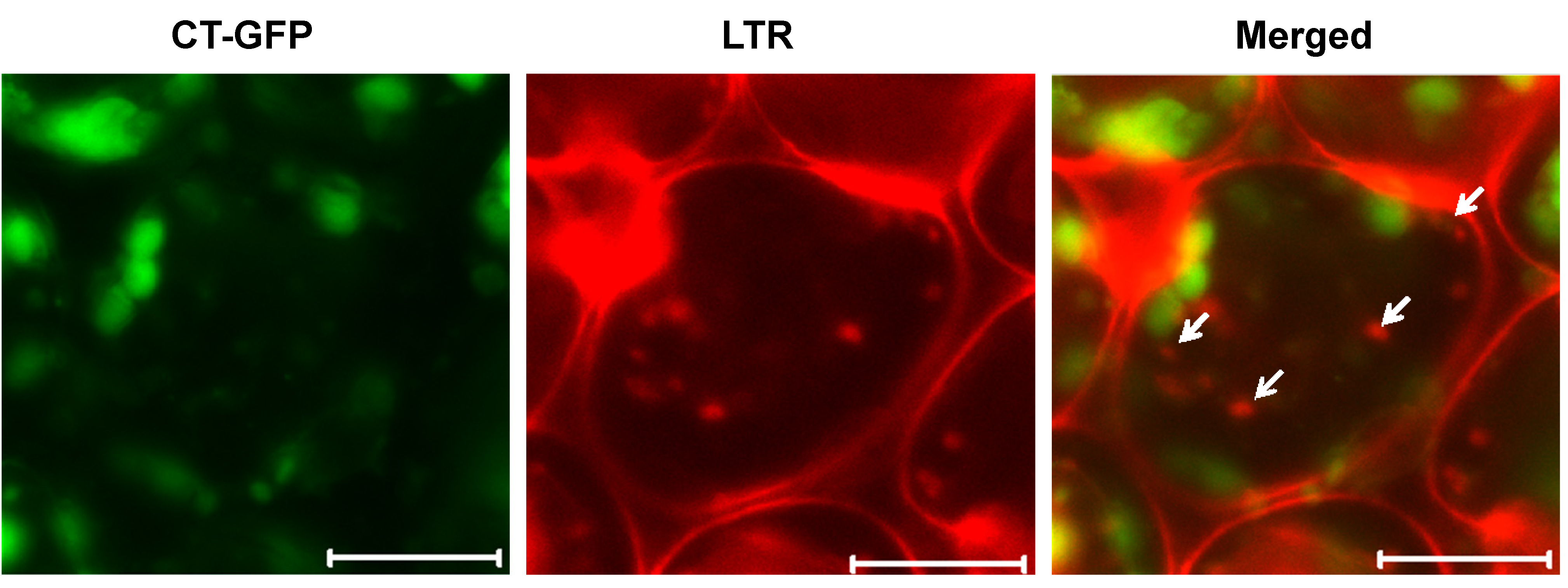

Supplement: Figure S2 — Visualization of CT-GFP and LTR staining of autophagosomal-related structures in mesophyll cells of Arabidopsis by LSCM. (TIF) [file pone.0073091.s002.tif]

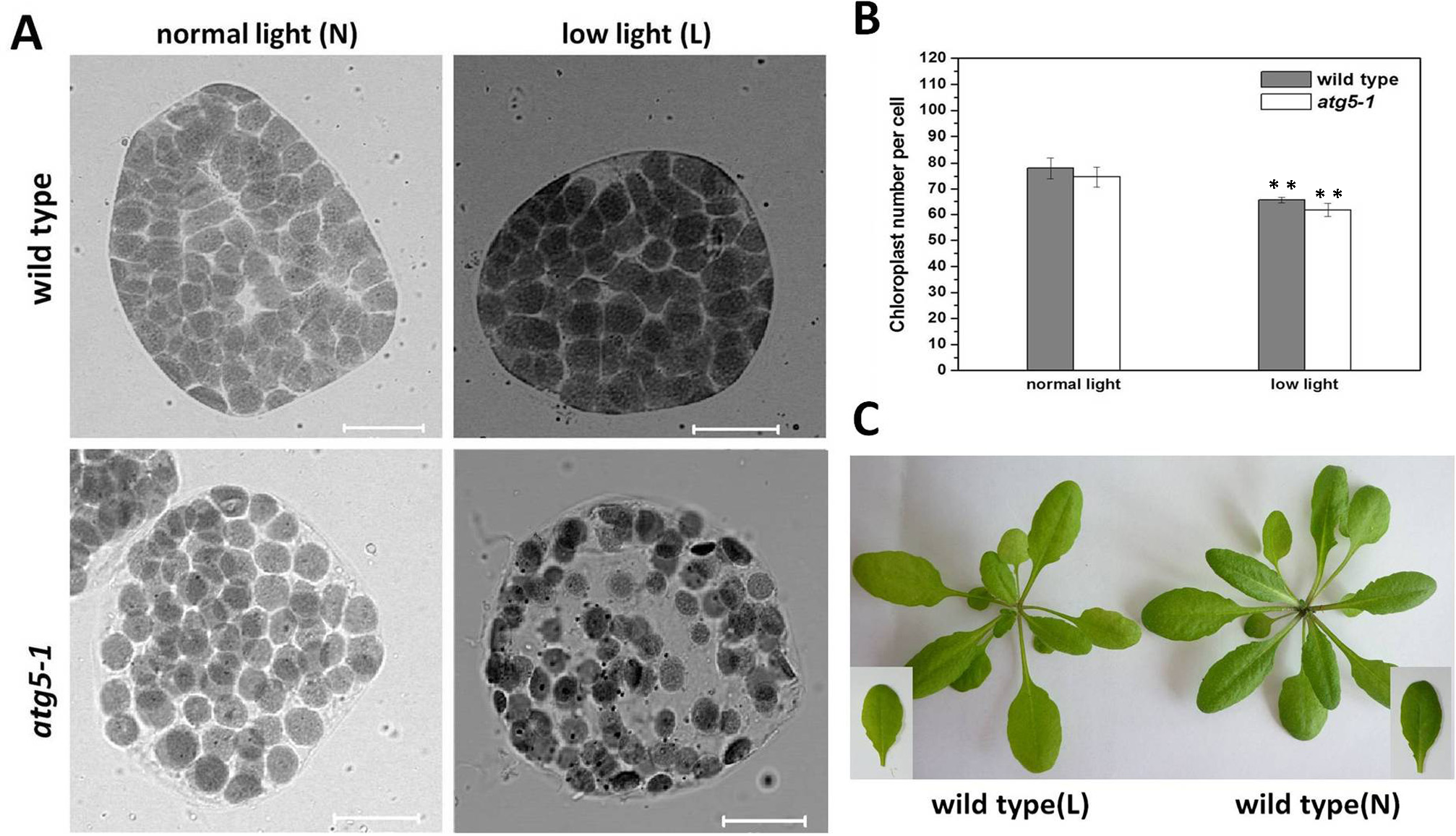

Supplement: Figure S3 — Differential interference contrast images of chloroplasts in mesophyll cells removed from leaves of wild-type and atg5-1 plants (A) and photographs of leaves of wild-type and atg5-1 plants (B and C). The wild-type and atg5-1 plants were respectively grown in a plant growth chamber with normal light and low light period for 3 weeks. (TIFF) [file pone.0073091.s003.tiff]

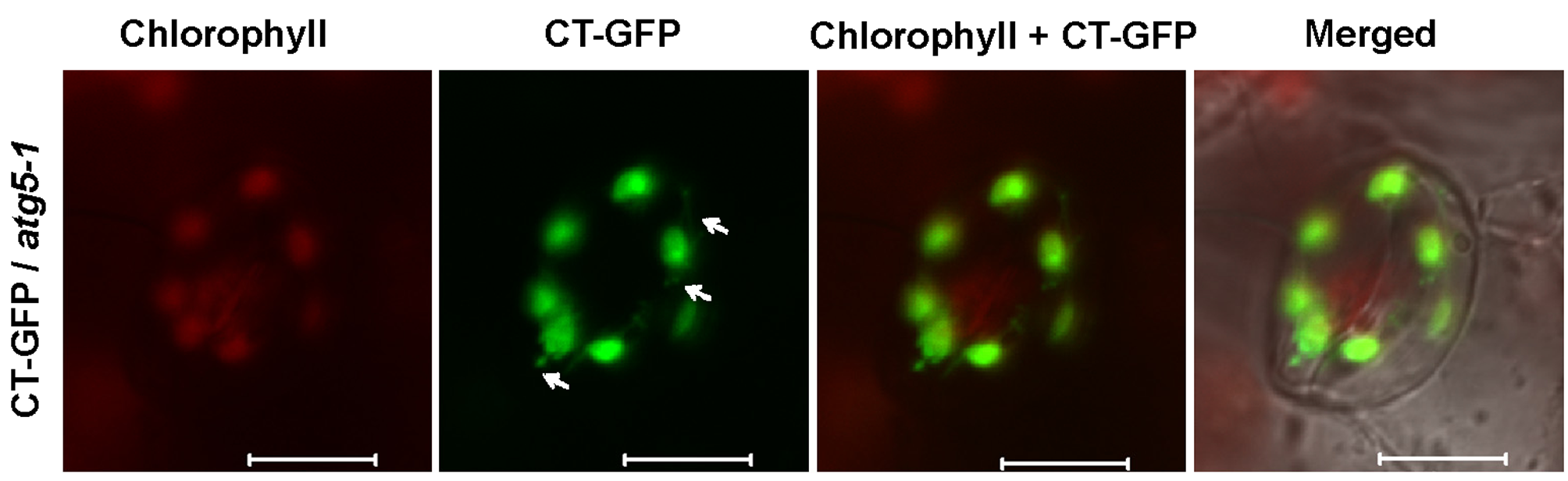

Supplement: Figure S4 — Visualization of the CT-GFP transgenic atg5-1 plant structures in guard cell of Arabidopsis by LSCM. (TIF) [file pone.0073091.s004.tif]
